# Associations of wildfire smoke PM_2.5_ exposure with cardiorespiratory events in Colorado 2011–2014

**DOI:** 10.1016/j.envint.2019.105151

**Published:** 2019-09-11

**Authors:** Jennifer D. Stowell, Guannan Geng, Eri Saikawa, Howard H. Chang, Joshua Fu, Cheng-En Yang, Qingzhao Zhu, Yang Liu, Matthew J. Strickland

**Affiliations:** a Department of Environmental Health, Rollins School of Public Health, Emory University, 1518 Clifton Road NE, Atlanta, GA 30322, USA; b Department of Environmental Sciences, Emory University, 201 Dowman Drive, Mailstop 1131-002-1AA, Atlanta, GA 30322, USA; c Department of Biostatistics and Bioinformatics, Rollins School of Public Health, Emory University, 1518 Clifton Road NE, Atlanta, GA 30322, USA; d Department of Civil and Environmental Engineering, University of Tennessee Knoxville, 851 Neyland Drive, Knoxville, TN 37996, USA; e School of Community Health Sciences, University of Nevada Reno, 1664 N. Virginia Street, Reno, NV 89557, USA

**Keywords:** Particulate matter, Wildfire, Air quality, Epidemiology, Climate change, Respiratory, Cardiovascular

## Abstract

**Background:**

Substantial increases in wildfire activity have been recorded in recent decades. Wildfires influence the chemical composition and concentration of particulate matter ≤2.5 μm in aerodynamic diameter (PM_2.5_). However, relatively few epidemiologic studies focus on the health impacts of wildfire smoke PM_2.5_ compared with the number of studies focusing on total PM_2.5_ exposure.

**Objectives:**

We estimated the associations between cardiorespiratory acute events and exposure to smoke PM_2.5_ in Colorado using a novel exposure model to separate smoke PM_2.5_ from background ambient PM_2.5_ levels.

**Methods:**

We obtained emergency department visits and hospitalizations for acute cardiorespiratory outcomes from Colorado for May-August 2011–2014, geocoded to a 4 km geographic grid. Combining ground measurements, chemical transport models, and remote sensing data, we estimated smoke PM_2.5_ and non-smoke PM_2.5_ on a 1 km spatial grid and aggregated to match the resolution of the health data. Time-stratified, case-crossover models were fit using conditional logistic regression to estimate associations between fire smoke PM_2.5_ and nonsmoke PM_2.5_ for overall and age-stratified outcomes using 2-day averaging windows for cardiovascular disease and 3-day windows for respiratory disease.

**Results:**

Per 1 μg/m^3^ increase in fire smoke PM_2.5_, statistically significant associations were observed for asthma (OR = 1.081 (1.058, 1.105)) and combined respiratory disease (OR = 1.021 (1.012, 1.031)). No significant relationships were evident for cardiovascular diseases and smoke PM_2.5_. Associations with non-smoke PM_2.5_ were null for all outcomes. Positive age-specific associations related to smoke PM_2.5_ were observed for asthma and combined respiratory disease in children, and for asthma, bronchitis, COPD, and combined respiratory disease in adults. No significant associations were found in older adults.

**Discussion:**

This is the first multi-year, high-resolution epidemiologic study to incorporate statistical and chemical transport modeling methods to estimate PM_2.5_ exposure due to wildfires. Our results allow for a more precise assessment of the population health impact of wildfire-related PM_2.5_ exposure in a changing climate.

## 1. Introduction

Climate change, defined as the long-term change in global and regional weather patterns, has been extensively documented since the mid-to-late 20th century (Boudes, 2011; Incropera, 2016; The Environmental Pollution Panel, 1965; United States Environmental Protection Agency, 2017b). Despite politically charged debates regarding the cause of the change, it is clear that climate change and its resulting extreme weather events could severely impact the health and well-being of populations across the globe (Berrang-Ford et al., 2015; Kjellstrom et al., 2016; Thornton et al., 2014; Wu et al., 2016). One area that reflects the synergistic impact of climate change and human activity is the occurrence of wildfires. Notably, the Western US has seen consistent and rapid increases in wildfire activity since the 1980s. This increase has been characterized by rises in the frequency, severity, size, and total burned area associated with wildfires (Liu et al., 2013; Westerling, 2016; Westerling et al., 2006). Fire effects are often seen at great distances from the events due to large smoke plumes, sometimes extending across multiple counties or states. States in the Rocky Mountain region continue to exhibit climatic factors conducive to fire activity—including high temperatures, low soil moisture, decreased rainfall, and increased solar radiation (Crockett and Westerling, 2018; Dawson et al., 2014; Griffin and Anchukaitis, 2014; Leung and Gustafson, 2005; Penrod et al., 2014). Conditions may become more suitable to large wildfires over time due to climate change (Keeley and Syphard, 2016; Keywood et al., 2013; Stavros et al., 2014). Consequently, wildfires place significant burdens on the human, economic, and environmental systems in areas surrounding and downwind from the burn zone. This is of particular concern given the impact that wildfire events can have on regional air quality and, subsequently, human health (Liu et al., 2015; Reid, Jerrett et al., 2016).

Wildfire smoke can produce significantly higher exposures to harmful compounds than are normally found in non-fire urban settings (Alves et al., 2011; Kim et al., 2018; Na and Cocker, 2008). Fine particulate matter (PM_2.5_, airborne particles < 2.5 μm in aerodynamic diameter) is of particular concern due to its ability to travel deep into the human respiratory system and enter the blood stream (Dockery and Pope, 1994; Hong et al., 2017; Kim et al., 2015; Liu et al., 2015; Park and Wexler, 2008; Reid, Jerrett et al., 2016; United States Environmental Protection Agency, 2017a). Smoke particles differ in both size and composition from particles found in typical ambient PM from non-wildfire sources. It has been shown that organic compounds, such as methanol or formaldehyde, make up a significantly higher proportion of smoke PM_2.5_ when compared with ambient PM (X.X. Liu, 2017; Na and Cocker, 2008). These distinctions could have differing effects on human health outcomes and may vary by fuel source. This has been shown in both in vivo and in vitro studies using human cells and mice (Kim et al., 2019; Shin et al., 2017; Xu et al., 2019). While much is left to be understood about the toxicological differences, current literature has begun to elucidate potential differences between smoke and ambient PM sources. It is, therefore, important to differentiate between smoke and non-smoke PM_2.5_ when assessing the health impact of wildfires.

While numerous epidemiological studies have established the associations between ambient PM_2.5_ and human health (Brook et al., 2010; Di et al., 2017; Pope and Dockery, 2006), relatively few studies have focused specifically on wildfire smoke (Rappold et al., 2017). For example, Reid et al. published a study showing a significant results for asthma during fire events (previous 2-day moving average) for a 5 μg/m^3^ change in PM_2.5_ concentration (Reid, Jerrett et al., 2016). While Reid et al. included satellite and chemical transport data, they were limited to the use of fire day and fire distance parameters to account for smoke PM instead of directly estimating smoke PM concentrations. Additionally, many studies are restricted to the use of ambient urban air pollution measurements, coupled with fire day indicators, to represent fire-related exposures. In addition, current guidelines for public health response to wildfire events rely heavily on changes of ambient total PM measurements due to a lack of information in wildfire-specific air quality (Lipsett et al., 2016). A few studies have distinguished among sources on larger scales (Hutchinson et al., 2018; J.C. Liu, 2017; Thelen et al., 2013). For example, Liu et al. derived metrics of smoke waves for distinguishing fire activity and evaluated the health impacts of smoke PM_2.5_ (J.C. Liu, 2017). Their chemical transport model simulations, however, were on a spatial grid of 0.5 × 0.67 degrees, which may be too coarse to capture finer-scale spatial gradients of exposure, see Supplemental Fig. 1.

Though there is consistent evidence for associations between wildfire events and disease, questions remain regarding the relationship between wildfire smoke PM_2.5_ and both respiratory and cardiovascular outcomes given the difficulty in estimating smoke PM_2.5_ exposure. Developing robust methods for understanding this complex relationship is vital to understand the potential future impacts of climate and wildfire events on human health. Building upon previous studies, the goal of our study is to estimate the associations for multiple respiratory and cardiovascular acute health events in relation to wildfire smoke PM_2.5_ in Colorado during the fire seasons of 2011–2014 using novel, high-resolution methods to separate wildfire smoke PM_2.5_ from background ambient PM_2.5_.

## 2. Methods

### 2.1. Health data

We obtained individual-level health data on daily hospitalizations and emergency department (ED) visits at all public and private hospitals for the fire seasons (May-August) of 2011–2014 from the Colorado Department of Public Health and Environment. Information included in the patient records are dates of admission, residential address, age, sex, payer information and International Classification of Diseases version 9 (ICD9) codes for primary and secondary diagnoses. Patients admitted to the hospital through the ED were only counted once, and those with elective hospitalizations were excluded from analysis.

We analyzed multiple endpoints for primary cardiovascular and respiratory diagnoses. Respiratory outcomes include asthma (ICD9: 493), bronchitis (ICD9: 490), chronic obstructive pulmonary disease (ICD9: 491, 492, and 496), upper respiratory infection (ICD9: 460–465 and 466.0), and combined respiratory disease (ICD9: 460–465, 466.0, 466.1, 466.11, 466.19, 480–486, 487, 488, 490, 491, 492, 496, and 493). Cardiovascular outcomes include ischemic heart disease (ICD9: 410–414), acute myocardial infarction (ICD9: 410), congestive heart failure (ICD9: 428), dysrhythmia (ICD9: 427), peripheral/cerebrovascular disease (ICD9: 433–437, 440, 443, 444, 451–453), and combined cardiovascular disease (ICD9: 410–414. 427, 428, 433–437, 440, 443, 444, 451–453). Due to inadequate numbers, events in children were not analyzed for COPD or any cardiovascular outcomes.

### 2.2. PM2_.5_ and meteorological data

We sought to separate smoke PM_2.5_ from ambient sources. To accomplish this, daily mean PM_2.5_ concentrations were adopted and improved from our previous study by adding new data (Geng et al., 2018). Briefly, mean concentrations were estimated using a two-model approach to combine information from high-resolution satellite AOD derived from the Multi-angle Implementation of Atmospheric Correction (MAIAC) algorithm, model simulations from the Community Multiscale Air Quality Modeling System (CMAQ), and ground measurements obtained from the U.S. Environmental Protection Agency (USEPA) for fire seasons in the state of Colorado (April-September 2011–2014). The first model (i.e. AOD model) utilized random forest modeling to incorporate MAIAC AOD, smoke mask, meteorological fields and land-use variables. The second model (i.e. CMAQ model) utilized statistical downscaling to calibrate the CMAQ PM_2.5_ simulations. Additional exposure modeling specifics can be found in Supplemental 2 and Supplemental Fig. 2. The output exposure data have full coverage in space and time and are able to capture the large fire events at a resolution of 1 km × 1 km (CV R^2^ = 0.81 and RMSE = 1.85 μg/m^3^). Compared to Geng et al. (2018), major improvements include new observation data from the National Park Service to capture PM_2.5_ enhancement near wildfires, allowing for a better representation of high values found during fire events (Supplemental 2 and Supplemental Fig. 2) (Benedict et al., 2017; Martin et al., 2013). Additionally, a random forest approach was utilized instead of the original statistical downscaler for the AOD model. This improved the R^2^ of the AOD model from 0.65 to 0.92 and the gap-filled R^2^ from 0.66 to 0.81 (Geng et al., 2018). PM_2.5_ exposure values were then aggregated to a 4 km × 4 km grid to match the resolution of the health data.

Fire count data were obtained using the MODIS fire count product to specify fire days for each grid cell (NASA, 2018). Wildfire and prescribed fire emissions were obtained from the US EPA emissions inventory for the study period. To calculate the wildfire smoke PM_2.5_ fractions, we used two CMAQ model scenarios-with and without smoke and dust particles. The differences between these scenarios were then divided by the total PM_2.5_ scenario to calculate the smoke PM_2.5_ fractions. The smoke PM_2.5_ fractions were then multiplied by the total satellite-based PM_2.5_ exposure to get the smoke PM_2.5_ concentrations.

### 2.3. Epidemiological modeling methods

We estimated associations between short-term changes in air quality and ED visits and hospital admissions using a case-crossover study design (Maclure, 1991). Each individual’s event day (i.e., date of ED visit or hospitalization) was matched with up to four non-event days, with matching based on grid location, day of week, and calendar month (Levy et al., 2001). Exposure and meteorology were assigned to each event day and corresponding non-event days based on the 4 km × 4 km grid cell in which the patient’s address is located. The 4 km grid was chosen a priori through collective agreement between the researchers and the Colorado State Health Department. This resolution was deemed the finest resolution we could use while still conserving confidentiality. We then used conditional logistic regression to estimate the associations between ED visits and hospitalizations for each outcome and exposure to non-smoke PM_2.5_ and smoke PM_2.5_. The final models for respiratory outcomes are shown in model specification 1 & 2 below:

(1) $$\mathrm{logit}\;P\left(Y\right)=\beta \left({\mathrm{total}}_{3\mathrm{day}}\;{\mathrm{PM}}_{2.5}\right)+\beta \left({\mathrm{temp}}_{3\mathrm{day}}\right)+\mathrm{ns}\left(\mathrm{doy}\right)$$

(2) $$\mathrm{logit}\;P\left(Y\right)=\beta \left({\mathrm{smoke}}_{3\mathrm{day}}{\mathrm{PM}}_{2.5}\right)+\beta \left({\mathrm{nonsmoke}}_{3\mathrm{day}}\;{\mathrm{PM}}_{2.5}\right)+\beta \left({\mathrm{temp}}_{3\mathrm{day}}\right)+\mathrm{ns}\left(\mathrm{doy}\right)$$

where *total_3day_* *PM_2.5_* represents the 3-day moving average for total PM_2.5_ (i.e., smoke + non-smoke), *temp_3day_* is the 3-day moving average temperature, *ns(doy)* is a spline for day of year (two internal nodes per year), *smoke_3day_* *PM_2.5_* represents the three-day moving average smoke PM_2.5_; and *nonsmoke_3day_* *PM_2.5_* denotes three-day moving average PM_2.5_ not related to wildfires. Cardiovascular outcome models were conducted using the same models shown in model specifications 1 and 2, but with 2-day averaging windows. Exposure windows of 3-day average PM for respiratory outcomes and 2-day average PM for cardiovascular outcomes were decided a priori based on published studies and consensus information found in the latest Integrated Science Assessment from the USEPA (Analitis et al., 2012; Delfino et al., 2009; Kunzli et al., 2006; J.C. Liu, 2017; Rappold et al., 2011; Reid, Jerrett et al., 2016; Strickland et al., 2010; USEPA, 2019). Sensitivity analyses were conducted using lag 0, lag 0–1 and seven-day exposure windows for respiratory outcomes and lag 0 and three-day exposure windows for cardiovascular outcomes.

Other potential confounders were assessed (relative humidity, boundary layer height, heat index, wind speed). However, these parameters did not influence the results and were omitted in the final model. Analyses to examine the presence of potential effect modification were completed using sex and age-stratification. Age-stratified categories included children (0–18 years), adults (19–64 years), and older adult (65+ years). We conducted all analyses in R 3.4.3 (2017) and SAS© 9.4.

## 3. Results

### 3.1. Exposure modeling and smoke contribution to *PM_2.5_* levels

**Fig. 1.**
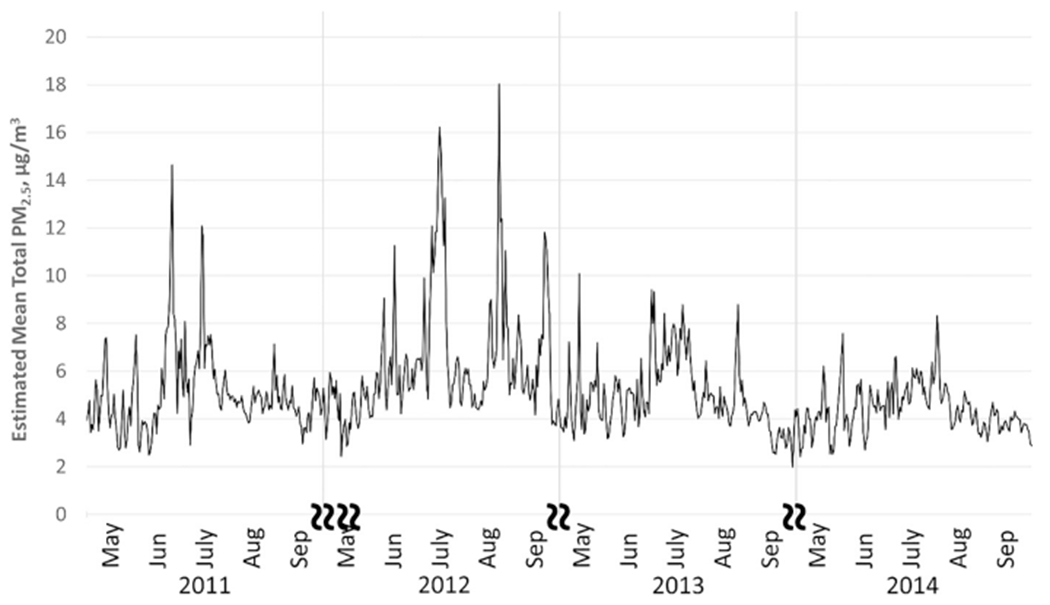
Daily mean modeled PM_2.5_ from for fire seasons 2011–2014 in Colorado. State-averaged time series data for fire seasons (May-August) 2011–2014 show total modeled PM_2.5_ levels by day, month, and year.

**Fig. 2.**
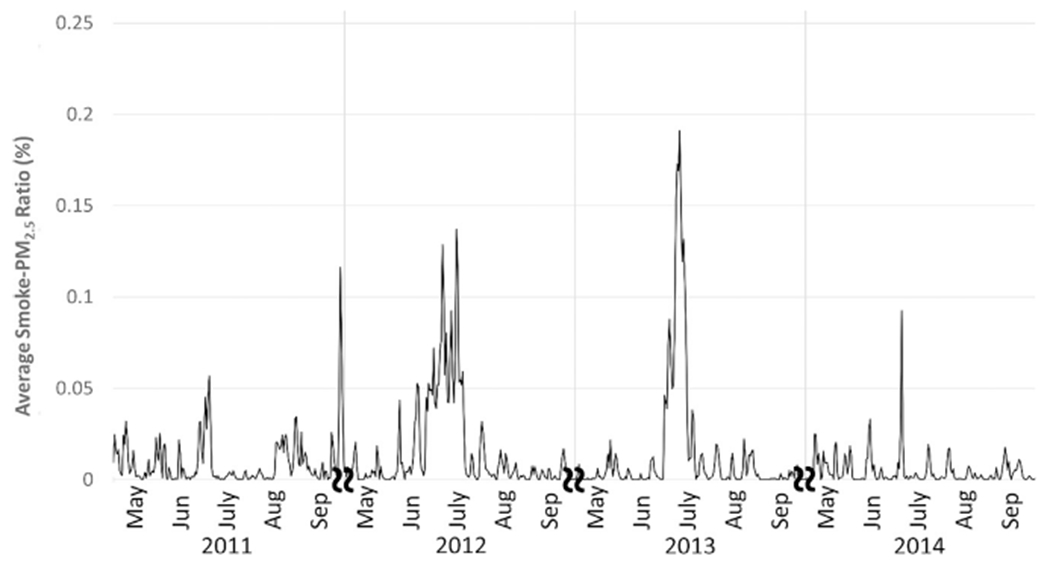
Daily mean ratio of PM_2.5_ attributed to wildfire. State-averaged time series data for fire seasons (May-August) 2011–2014 depicting ratio of modeled smoke PM_2.5_ to total modeled PM_2.5_.

**Fig. 3.**
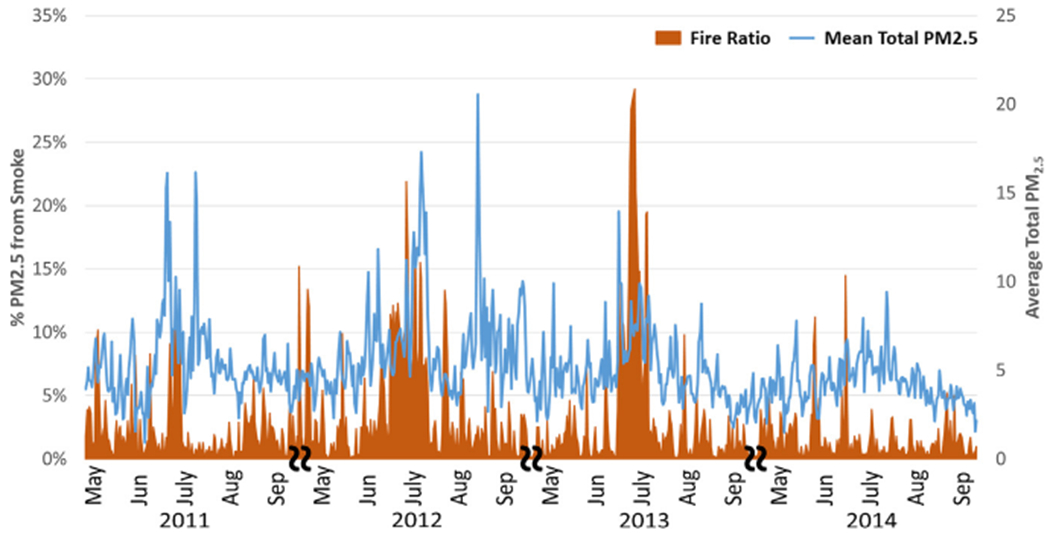
Domain-wide daily mean total PM_2.5_ and mean ratio of PM_2.5_ on fire days (fire PM > 1%). Time series depicting both total and ratio of modeled smoke PM_2.5_ to total modeled PM_2.5_.

**Fig. 4.**
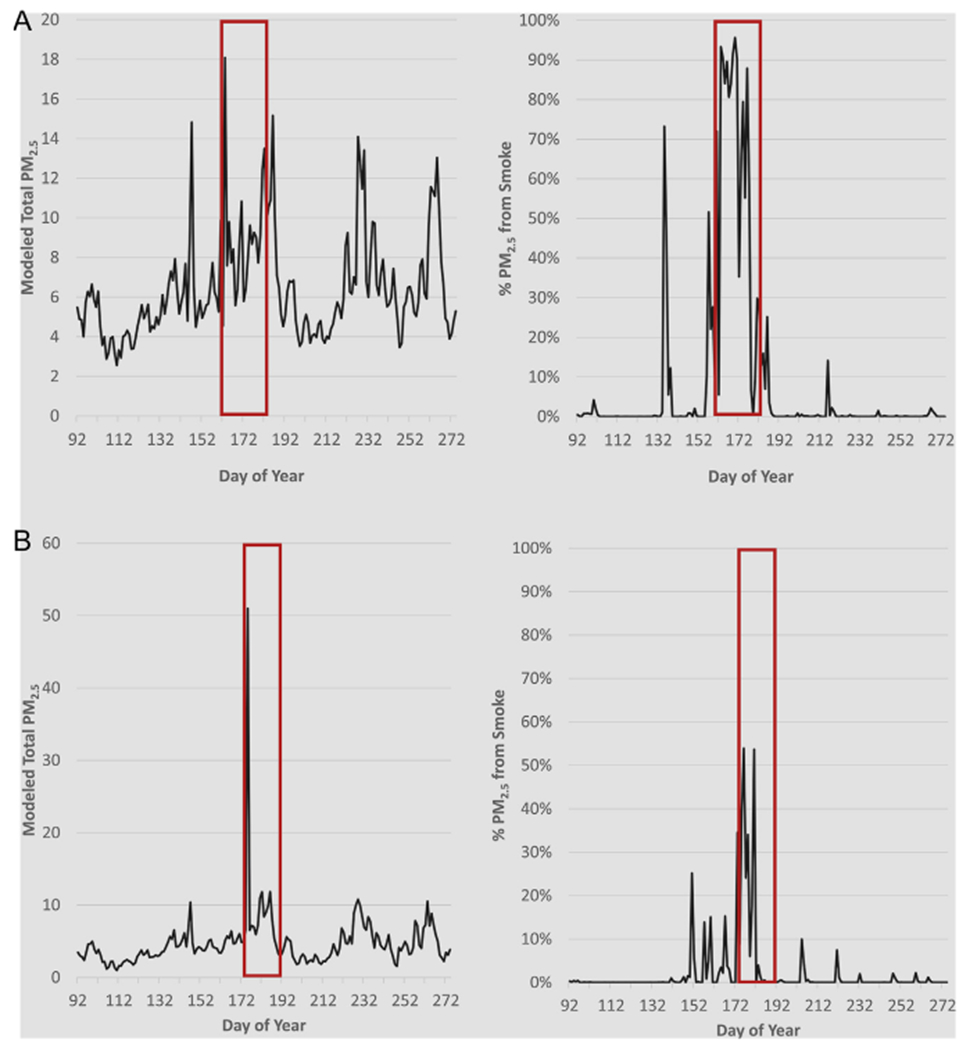
Daily mean total PM_2.5_ and mean ratio of PM_2.5_ attributed to wildfire at two locations. Time series depicting both total and ratio of modeled smoke PM_2.5_ to total modeled PM_2.5_. A) Location near the High Park Fire (June 9–30, 2012) and B) Location near Waldo Canyon Fire (June 23-July 10, 2012). Red boxes indicate active fire days. (For interpretation of the references to color in this figure legend, the reader is referred to the web version of this article.)

**Fig. 5.**
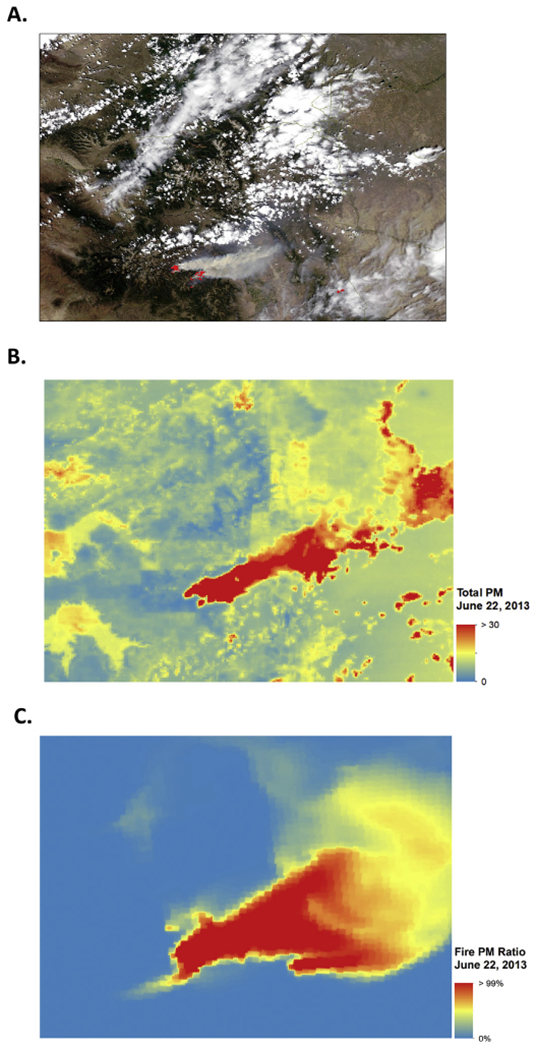
Satellite smoke plume, modeled total PM_2.5_ and smoke PM_2.5_ for west fork fire complex, June 22, 2013. Modeled data corresponds to visible smoke plume as shown in A-C. A) Satellite image from June 22, 2013 with active West Fork Complex Fire (NASA, 2013). B) Total PM_2.5_ for Colorado on June 22, 2013. C) Amount of PM_2.5_ attributed to fire on June 22, 2013.

A time series plot for modeled statewide daily mean PM_2.5_ concentrations is shown in Fig. 1. Modeled total PM_2.5_ values ranged from close to 0 to 47.48 μg/m^3^, with an overall mean value of 4.67 μg/m^3^. The exposure model was also used to separate smoke PM_2.5_ from nonsmoke PM_2.5_. This separation is based on the CMAQ fraction, with total PM_2.5_ equal to the sum of non-smoke PM_2.5_ and smoke PM_2.5_. Ratios of smoke PM_2.5_ to total PM_2.5 _ ranged from 0 to 99.56% (mean = 0.006%), with smoke PM_2.5_ levels ranging from 0 to 37.34 μg/m^3^. The statewide daily mean smoke vs. total PM_2.5_ ratio is also shown for the entire study period (See Fig. 2). As shown, concentrations varied year-to-year and between stations. This is likely due to the spatial variability of wildfires and varied smoke plume behavior due to factors such as prevailing wind speed and direction. To illustrate PM2.5 concentrations and ratios attributable to fire, Fig. 3 shows the domain-wide average total PM2.5 on fire days (smoke PM2.5 > 1%) compared with the domain-wide average ratio of smoke PM2.5. For the entire study period, total PM2.5 averaged 7.87 μg/m3 with average fire PM2.5 ratios at 28%. Fig. 4 shows locations on a fire day near two major fires that occurred during our study period. As shown in Fig. 4A, high levels of smoke PM can be seen despite more moderate total PM2.5 concentrations. Fig. 4B depicts a fire day with much higher total PM2.5 concentrations and the subsequent contributions of smoke PM. Additional analysis showed relatively little correlation between smoke PM_2.5_ and non-smoke PM_2.5_ (Pearson correlation coefficient r = 0.11, p < 0.0001). The peaks of highest smoke PM_2.5_ ratios tended to correspond with active fire days. Fig. 5 illustrates the modeled total PM_2.5_ and smoke PM_2.5_ ratio for June 22, 2013, a peak fire day during the West Fork Fire Complex. As depicted, when compared to satellite imaging, the modeled smoke PM_2.5_ appears to capture the apparent visible smoke plume adequately.

### 3.2. Epidemiological modeling

After excluding duplicate events and events with non-geocoded addresses, 44,262 of 490,368 (9%) of cases were excluded from the analysis. A total of 446,106 ED visit and hospitalization events were analyzed from the Colorado Department of Public Health and Environment. Of those included, there were 204,823 male and 241,283 female cases. The lowest case count occurred in 2011 (n = 102,318), with the highest number of cases in 2014 (n = 129,477). While many reasons could exist, the large increase seen in 2014 could be explained by changes in health seeking behavior due to wider Medicaid coverage resulting from the implementation of the Affordable Care Act (Singer et al., 2019). Other summary statistics on age groups and events per year are found in Table 1.

**Fig. 6.**
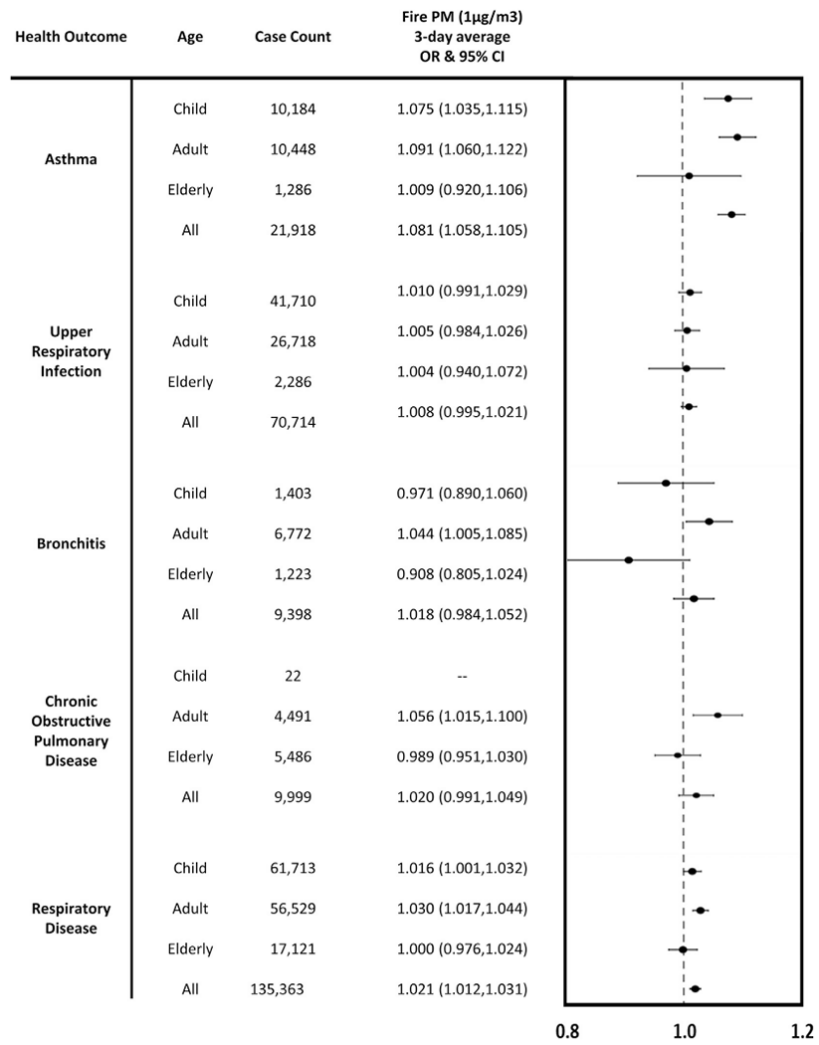
Wildfire smoke PM_2.5_ exposure and respiratory outcomes. Odds ratios for both total and age-stratified respiratory outcomes per 1 μg/m^3^ increase in wildfire smoke PM_2.5_ exposure, arranged by outcome and age group.

**Fig. 7.**
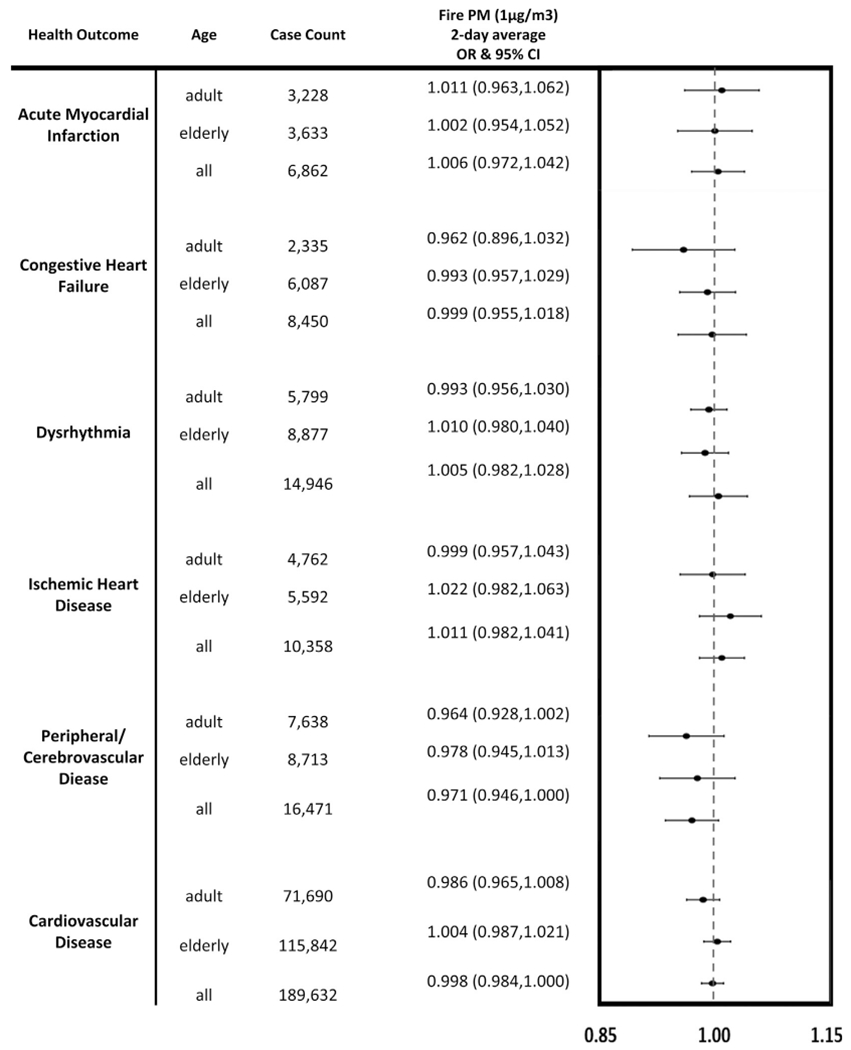
Wildfire smoke PM_2.5_ exposure and cardiovascular outcomes. Odds ratios for both total and age-stratified cardiovascular outcomes per 1 μg/m^3^ increase in wildfire smoke PM_2.5_ exposure, arranged by outcome and age group.

Using conditional logistic regression models, we estimated the odds ratio for exposure to smoke PM_2.5_ and individual health outcomes. As shown in Fig. 6 and Supplemental Table 1, we observed significant positive associations between 1 μg/m^3^ increases in 3-day moving average fire exposures and both asthma (OR 1.081, 95% CI (1.058, 1.105)) and combined respiratory disease (OR 1.021, 95% CI (1.012, 1.031)) in a model that adjusted for PM_2.5_ from other sources. There were no significant positive associations linked to cardiovascular outcomes and 2-day smoke PM_2.5_ exposures (see Fig. 7 and Supplemental Table 2). However, some inverse associations were shown to be protective for cardiovascular outcomes. This could possibly be due to random error, or it may be that individuals with pre-existing cardiovascular disease stay indoors on days with fire activity.

The models were also run using total PM_2.5_ for both cardiovascular and respiratory outcomes. Overall, the majority of the respiratory odds ratios for 3-day average total PM_2.5_ were either null or trending to positive (Supplemental Table 3).The odds ratios for ischemic heart disease, acute myocardial infarction, and dysrhythmia also suggest a trend toward a positive association (see Supplemental Table 4). The cardiovascular results for total PM_2.5_ included significant negative results for congestive heart failure, peripheral/cerebrovascular disease, and cardiovascular disease.

We conducted sensitivity analyses for additional exposure windows. Using lag 0 for both respiratory and cardiovascular outcomes, similar results were seen with smoke PM_2.5_ exposure, with notable differences in overall upper respiratory infection (OR 1.015, 95% CI (1.005, 1.026)) and upper respiratory infection in children (OR 1.018, 95% CI (1.004, 1.003), see Supplemental Figs. 3 and 4). Using lag 0–1 for all respiratory outcomes, the results were again similar to the initial analysis with changes for overall and child-only upper respiratory infections; see Supplemental Fig. 5. Using a 7-day averaging window for respiratory outcomes, asthma was the only outcome to have a significant positive association with smoke PM_2.5_ exposure (OR 1.081, 95% CI (1.051, 1.112), see Supplemental Table 5). The associations for asthma, upper respiratory infection, bronchitis, and combined respiratory disease trended positive but not significant for 7-day averaged total PM_2.5_ exposure (see Supplemental Table 6). A 3-day averaging window used for cardiovascular outcomes also yielded either null or negative results (Supplemental Tables 7 and 8).

### 3.3. Stratified analysis

To investigate potential effect modification of the relationship between exposures and respiratory outcomes, we conducted stratified analyses based on sex and age. While most sex-stratified total PM_2.5_ results were null, an association was seen in females for bronchitis (OR 1.007, 95% CI (1.001, 1.013), see Supplemental Table 9), however, no significant results were observed for cardiovascular outcomes and both 2-day total and smoke PM_2.5_ (Supplemental Tables 10 and 11). Associations for both female and male asthma cases and 3-day average smoke PM_2.5_ were significant, with higher odds shown in female cases (OR 1.096, 95% CI (1.064, 1.128)) than in male cases (OR 1.063, 95% CI (1.029, 1.098)). Female bronchitis cases (OR 1.054, 95% CI (1.010, 1.101)) and female total respiratory cases (OR 1.027, 95% CI (1.015, 1.040)) were also positively associated with smoke PM_2.5_. Additional sex-stratified, 3-day average smoke PM_2.5_ results can be found in Supplemental Table 12.

Additionally, some outcomes exhibited differences when stratified on age. After age-stratification, there were no patterns found linking respiratory outcomes and total PM_2.5_ with any specific age group (Supplemental Table 13). Regarding smoke PM_2.5_, Fig. 6 also depicts the ORs and associated confidence intervals for each of the respiratory outcomes by age group. In children ages 0 to 18 years, significant positive associations were seen for asthma (OR 1.075, 95% CI (1.035, 1.116)). Adults aged 19 to 64 years of age exhibited positive associations for asthma (OR 1.091, 95% CI (1.060, 1.122)), bronchitis (OR 1.044, 95% CI (1.005, 1.085)), COPD (OR 1.056, 95% CI (1.015, 1.100)), and combined respiratory disease (OR 1.030, 95% CI (1.017, 1.044)) (see also Supplemental Table 14). For individuals 65 and older, there were no significant positive associations seen for respiratory outcomes. We found no positive associations for age-stratified total or smoke PM_2.5_ and any of the cardiovascular outcomes (See Fig. 7 and Supplemental Tables 15 and 16). Additional results for stratification analyses using a 7-day averaging window for respiratory outcomes and a 3-day averaging window for cardiovascular outcomes can be found in Supplemental Tables 17–24. Of note, associations for both childhood and adult asthma, adult COPD, and adult combined respiratory disease events were positively associated with 7-day average smoke PM_2.5_ (see Supplemental Table 17).

## 4. Discussion

In this study, we estimated associations between various health outcomes and acute exposure to non-smoke PM_2.5_ and smoke PM_2.5_ in the state of Colorado over a four-year period (2011–2014). The design of this study is centered on smoke PM_2.5_ contributions to health outcomes. This work builds on our previous work by improving exposure data metrics and expanding from a 1-month pilot study (Alman et al., 2016). The exposure data considers both spatial and temporal variability by including the use of satellite data to enhance the exposure estimates on an improved spatial scale of 4 km × 4 km. Another unique aspect of our exposure assessment is that we were able to separate smoke PM_2.5_ from non-smoke sources and estimate risks attributable to wildfire smoke distinct from those due to PM_2.5_ exposures from other sources.

As we hypothesized, many of the respiratory disease outcomes increased during periods of wildfire activity. For respiratory outcomes, we estimated an increase (OR = 1.036 (95% CI: 1.022, 1.050%)) in ED/hospitalizations per 1 μg/m^3^ increase in fire smoke PM_2.5_ exposure. The magnitude of the association was largest for asthma (OR = 1.081 (95% CI: 1.058, 1.105)). Additionally, we observed heterogeneity in the association estimates when stratifying by age group. Positive associations were observed for asthma events, where ED/hospitalizations increased significantly in children (OR = 1.075 (95% CI: 1.035, 1.116)) and in adults (OR = 1.091 (95% CI: 1.060, 1.122)) whereas the association estimate was lower in magnitude and was less precise for older adults (OR = 1.009 (95% CI: 0.920, 1.106)). Similarly, an increase was seen for combined respiratory diseases with increases in ED/hospitalizations and adults (OR = 1.030 (95% CI: 1.017, 1.044)). Specifically, in the adult group, increases were also shown for both bronchitis (OR = 1.044 (95% CI: 1.005, 1.085)) and COPD (OR = 1.056 (95% CI: 1.015, 1.100)). As opposed to other studies, there was no association shown for respiratory diseases when stratified for the older adult age group.

Unlike respiratory outcomes, we did not see a strong link between smoke PM_2.5_ and cardiovascular outcomes. Results for combined cardiovascular disease yielded null results (OR = 0.998 (95% CI: 0.984, 1.011)). Similar results were shown for both the adult and older adult age groups. This is not wholly surprising given differing results in current literature regarding the links between cardiovascular outcomes and wildfire events. There are fewer examples of cardiovascular associations with wildfire smoke exposure compared to respiratory outcomes. Additionally, associations with cardiovascular outcomes tended to be substantially lower in magnitude than for the respiratory outcomes. These differences are consistent with published studies on both types of outcomes (Cascio, 2018; Deflorio-Barker et al., 2019; Dennekamp et al., 2011; Dennekamp et al., 2015; Johnston et al., 2014; Liu et al., 2015; Reid, Brauer et al., 2016; Wettstein et al., 2018). For example, in Deflorio-Barker et al. (2019), most cardiovascular outcomes were not significant with fire day PM2.5 using lag0-2. They also found similar results for smoke day all-cause cardiovascular outcomes were very similar to non-smoke days (OR 1.06 for smoke days vs OR 1.07 for non-smoke days (Deflorio-Barker et al., 2019)).

Our high-resolution epidemiological study furthers the current knowledge in the field by incorporating random forest modeling methods combining information from MAIAC AOD, CMAQ simulations, and ground measurements to elucidate the portion of PM2.5 present in the air due to wildfire smoke. Previous work has been done to enhance the spatial coverage and resolution of total PM2.5 estimates during wildfire events (Reid et al., 2019). While most work compared smoke and non-smoke days using various fire indicators, our study particularly focuses on the separation of smoke PM2.5 from other sources. In most work, researchers compared smoke and non-smoke days using a variety of methods different from our study (Reid et al., 2019; Reid, Jerrett et al., 2016). For example, satellite measurements are increasingly used to augment the spatially sparse ground monitoring for PM. However, this remains a relatively new approach to capturing the smoke PM concentrations. A study by Liu et al. looked at the entire Western US at the county-level using combined satellite and ground data (J.C. Liu, 2017). They defined a fire indicator variable, or “smoke wave,” which includes periods of at least two days of high pollution from wildfire smoke. Using this method, Liu et al. found associations between wildfire smoke exposure and various respiratory illnesses, but no associations with cardiovascular outcomes. Reid et al. (2015) used a machine learning approach to integrate multiple data sources including smoke indicators such as the distance to the nearest fire cluster and a smoke intensity calculation. The use of more advanced methods for predicting PM_2.5_ exposure enhanced the exposure estimations, however, the PM_2.5_ concentrations were not separated into smoke and non-smoke concentrations (Reid et al., 2015).

Other work has utilized methods combining wildfire emissions and smoke plume modeling. For example, Hutchinson et al. examined similar epidemiological questions using exposure data derived from a model that combined the Wildland Fire Emissions Information System and the Hybrid Single-Particle Lagrangian Integrated Trajectories (Hutchinson et al., 2018). Their study found increases in respiratory events with null cardiovascular results. However, the methods denoted fire-specific emissions due to fire location and progression from modeled progression maps and may not capture exposures as well as the use of chemical transport models. Ultimately, while our results carry similar interpretations to both studies, subtle dissimilarities may be seen as we utilize different air quality evaluation products and higher-resolution meteorological and epidemiological data to better-define the local exposures for each event.

The asthma association found in our study is substantially larger than those shown in previous publications. In addition to Reid, Brauer et al. (2016), other studies found significant associations between smoke PM and health outcomes. Delfino et al. reported significant associations of OR = 1.043 between asthma and 2-day moving average smoke exposure for 10 μg/m3 increase in total PM2.5 concentration (Delfino et al., 2009). In a more recent study, Reid et al. also found a significant association for asthma and previous 2-day moving average smoke exposure, with an OR of 1.050 during fire events for a 10 μg/m3 increase in PM2.5 (Reid et al., 2019). Factoring in the domain-wide average smoke PM2.5 ratio for the study period (~28% for days with > 1% smoke PM), our result per 1 μg/m3 roughly translates to 1.08 per 4 μg/m3 of total PM2.5. This converted result is more aligned with previously reported values, and the larger effect estimate is likely due to improved exposure assessment. It is also important to remember that our methods are unlike the majority of previous literature. Namely, the general approach in previous studies is to model smoke exposure using smoke day indicators. Our approach differed in that we sought to isolate the actual concentration of PM2.5 directly from smoke. We originally hypothesized that there may be a difference in toxicity of smoke PM2.5 compared to non-smoke PM2.5. When compared with other literature, our findings suggest that smoke PM2.5 may actually be more damaging to human health. Aside from asthma outcomes, the majority of the health associations in this study fall in line with those found in previous literature. For example, Deflorio-Barker et al. (2019) also demonstrated stronger associations with respiratory outcomes than those with cardiovascular disease; with asthma exhibiting the largest OR of 1.06 (Deflorio-Barker et al., 2019).

While we did not investigate physiological mechanisms, these results may be explained by the toxicity of smoke PM_2.5_. Since different chemical compositions of PM_2.5_ may affect the body differently, it has been suggested that toxicological differences may play a role in how wildfire smoke PM affects the human anatomy and physiology. Multiple toxicological studies have shown differences in the composition and effects of wildfire smoke compared to ambient air (Franzi et al., 2011; Kim et al., 2018; Wegesser et al., 2010; Wegesser et al., 2009; Wong et al., 2011). It has been shown that the small particles found in wildfire smoke may be responsible for stimulation of mechanisms that lead to increased oxidative stress at the cellular level. Wegesser et al. (2009) observed significant changes in macrophage and neutrophil counts in mouse lung samples exposed to wildfire smoke PM compared to ambient air. An additional study by the same group, expanded on these findings to show that substances such as polycyclic aromatic hydrocarbons (PAH) can be present in much higher concentrations in smoke versus levels detected in ambient air (Wegesser et al., 2010). Franzi et al., (2011) looked specifically at the inflammatory responses due to wildfire smoke PM exposure. PM from wildfire smoke exhibited approximately five times more toxicity to lung macrophages than nonsmoke exposure. This study also showed significant changes in reactive oxygen species and subsequent oxidative stress, leading to higher cell degeneration and potential apoptosis. Similarly, Kim et al. (2018) found significant increases in mouse lung neutrophils after exposure and that levels of lung toxicity were significantly associated with fuel type (Kim et al., 2018).

Despite the strengths of our study, some limitations remain. While we sought to enhance the exposure estimates for individual cases, some exposure misclassification is still possible given the assumption that the location of a person’s address is a good representation of their short-term exposures to smoke PM. An additional limitation exists due to the use of modeled exposure data. However, as stated previously and despite this uncertainty, the model accurately captures the temporal and spatial trends of PM_2.5_ measured by ground monitors and, thus give an accurate representation of overall trends. Additionally, several health events were left out of the analysis due to issues with address geocoding or non-Colorado residency. However, the exclusions were relatively small with only 9% of cases not used in the final analyses. Additionally, our analyses lacked the ability to differentiate chemical compositions of PM_2.5_. Thus, we cannot link toxicological effects to our exposure metrics. Finally, the selection of averaging window size, though based on current literature, may also introduce error into the analysis.

Notwithstanding these limitations, our methods lend insight into important challenges that remain in the wildfire smoke exposure and health effects literature. The use of higher resolution enhanced exposure data provides a new approach to assigning exposure to individual events. Using multiple data products, our method aids in distinguishing wildfire smoke PM_2.5_ from background PM_2.5_. Unlike ground monitors that provide spatially sparse measurements, the exposure model used here provides daily concentrations for each 4 km × 4 km grid cell in our epidemiological study.

## 5. Conclusions

Supported by high-resolution PM_2.5_ exposure estimates, we found significant associations between wildfire smoke and acute respiratory outcomes in Colorado, despite an absence of association with total PM_2.5_ concentrations. Our findings point to potential toxic differences between smoke and non-smoke PM_2.5_ exposure; suggesting that PM_2.5_ from wildfire smoke could pose a significant threat to public health. This is especially true given the expected climate change-related impacts on wildfire incidence. It is, therefore, important to derive more accurate concentration-response relationships specific to wildfire smoke in order to develop a better understanding of future potential health risks based on increased wildfire activity. Taken together, the current analysis can inform public health agencies and healthcare systems regarding the potential future burden of wildfire smoke PM_2.5_ exposure within the context of climate change. This information may be a key element in evaluating and enhancing current preparations aimed at wildfire-event response readiness.

## Supplementary Material

Supplementary data

## Figures and Tables

**Table 1:** Epidemiologic data descriptive statistics.

	Case count
Total records	490,368
Geocoded addresses	446,106
Non-geocoded addresses	44,262
Year of event	
2011	102,318
2012	102,574
2013	111,737
2014	129,477
Age ranges	
0–18 y	94,022
19–64 y	202,665
65+ y	149,419
Sex	
Female	241,282
Male	204,823
