# Supplementary material for: Associations of wildfire smoke PM2.5 exposure with cardiorespiratory events in Colorado 2011–2014
Source: Environ Int. Author manuscript; Available in PMC 2021 May 28. (PMC8163094; doi:10.1016/j.envint.2019.105151)
Supplement: Supplementary data [file NIHMS1699076-supplement-Supplementary_data.docx]

Supplemental Figure 1. Comparing 2012 PM_2.5_ annual average concentrations for A) coarse grid scale (0.50x0.67 degrees) and B) 4km study grid. As shown, the coarse resolution CTM results could not resolve the PM2.5 concentration gradients related to the rapid terrain change.

**B)**

**A)**


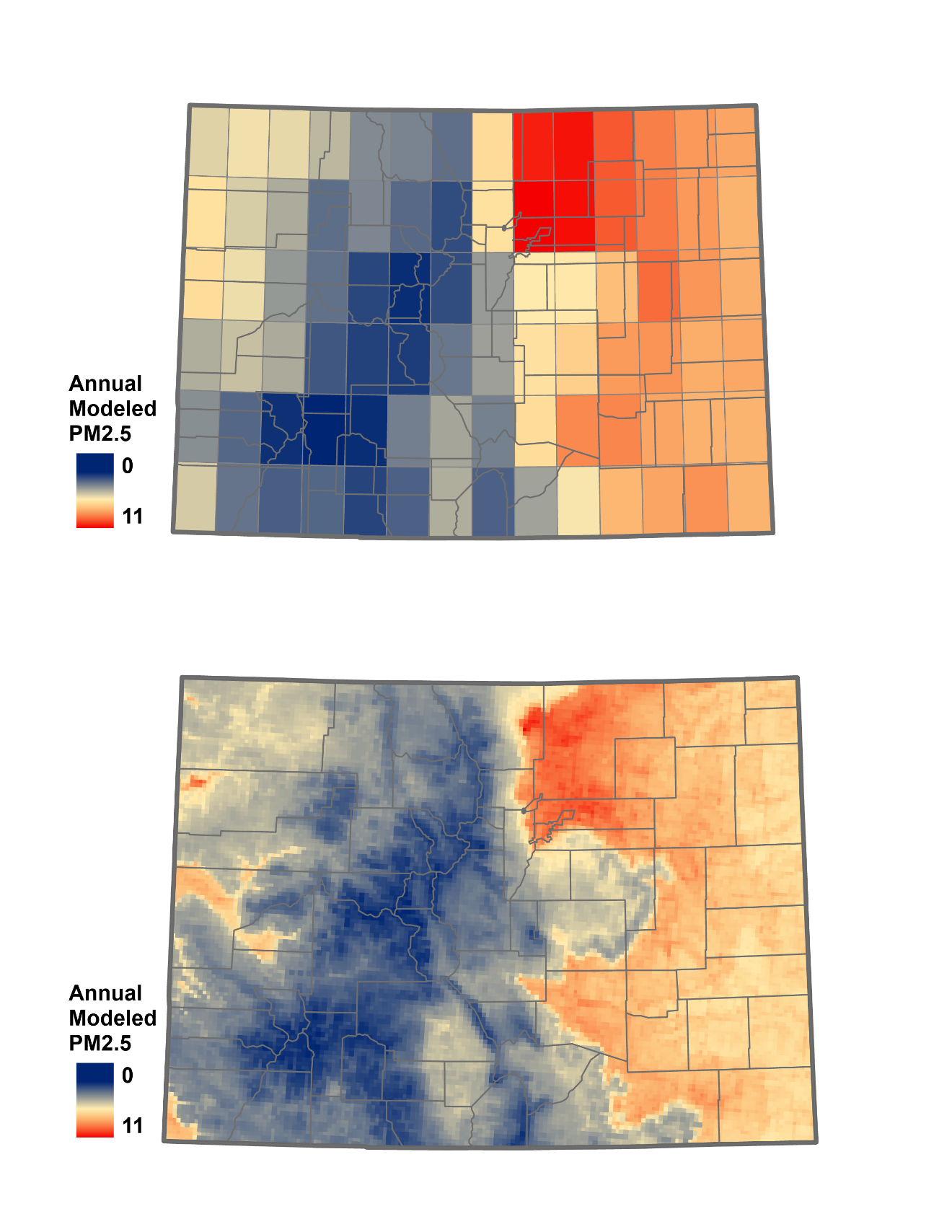


**Supplemental 2 PM_2.5_** **Exposure Modeling**

The PM_2.5_ estimates used in this study are based on our previous work (Geng et al., 2018), with the incorporation of some additional measurements. The input dataset, which includes ground PM_2.5_ observations, MAIAC AOD, smoke mask, meteorological fields, land-use variables and CMAQ simulations, are adopted directly from Geng et al. (2018). More details about the data can be found in Geng et al. (2018). To better reflect the PM_2.5_ enhancements during fire events, we also utilize PM_2.5_ measurements during May-June, 2012 from the National Park Service made at the Atmospheric Science Department of Colorado State University, which is near the edge of the 2012 High Park Fire (Val Martin et al., 2013; Benedict et al., 2017).

Two models are involved to provide a full spatial and temporal coverage of PM_2.5_ estimates. The first is a random forest model that incorporates MAIAC AOD, smoke mask, meteorological fields, and as land-use variables (i.e. the AOD model). The second is a statistical downscaler that calibrates the CMAQ PM_2.5_ simulations, as described in Geng et al. (2018). For grid cells that have missing values in the AOD downscaler due to the missing of MAIAC AOD, we use the estimated values from the CMAQ downscaler to fill in the gaps.

The out-of-bag R-squared of the AOD model is 0.92, and the 10-fold cross-validated (CV) R-sqaured of the CMAQ downscaler is 0.52. Overall, the CV R-squared is 0.81. The estimated PM2.5 data capture the elevated PM_2.5_ concentrations during fire events, as shown in an example in Supplemental Figure 1.


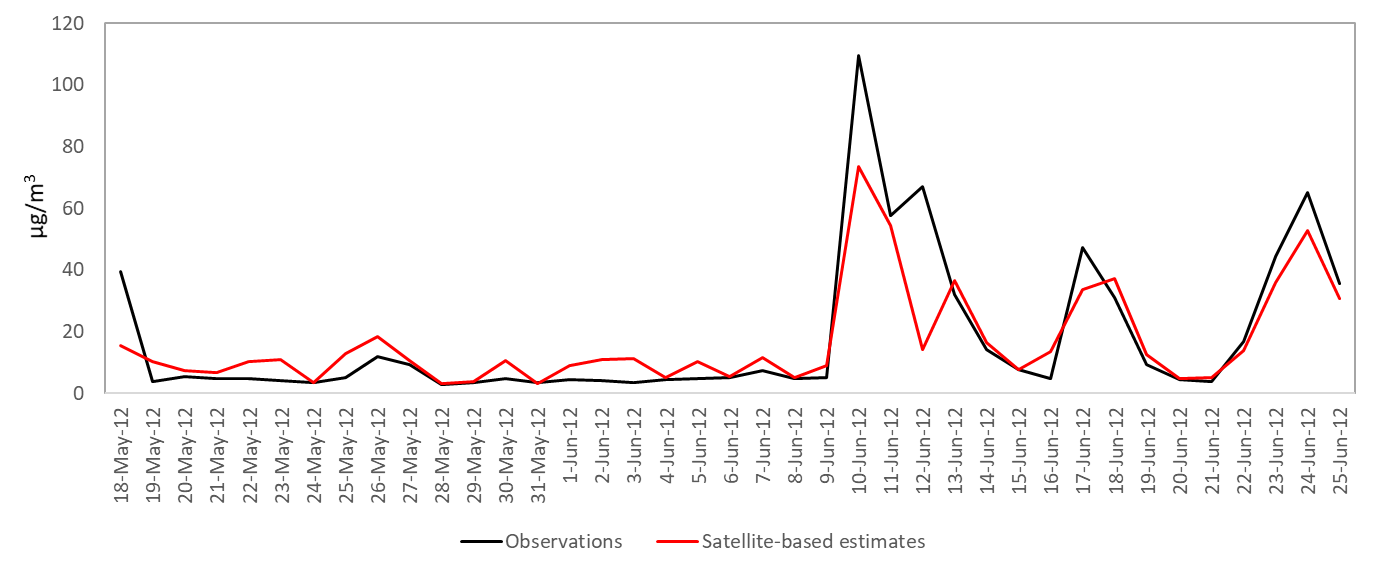


Supplemental Figure 2. Time series of PM_2.5_ observations and our estimates during High Park Fire in a near monitor

References:

Benedict, K.B., Prenni, A.J., Carrico, C.M., Sullivan, A.P., Schichtel, B.A., Collett, J.L., 2017. Enhanced concentrations of reactive nitrogen species in wildfire smoke. Atmospheric Environment 148, 8-15

Geng, G., Murray, N.L., Tong, D., Fu, J.S., Hu, X., Lee, P., et al., 2018. Satellite-Based Daily PM2.5 Estimates During Fire Seasons in Colorado. Journal of Geophysical Research: Atmospheres 123 (15), 8159-8171

Val Martin, M., Heald, C.L., Ford, B., Prenni, A.J., Wiedinmyer, C., 2013. A decadal satellite analysis of the origins and impacts of smoke in Colorado. Atmos Chem Phys 13 (15), 7429-7439


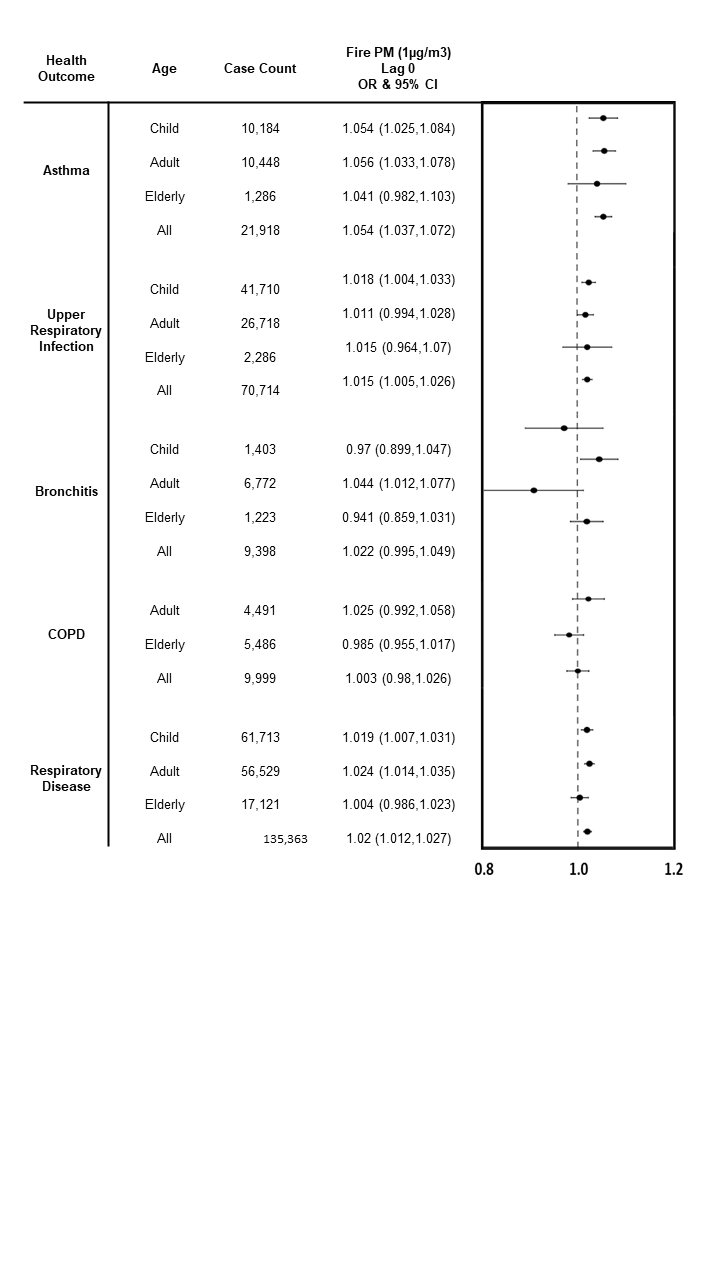


Supplemental Figure 3. OR Respiratory Results for Lag 0 Exposures.


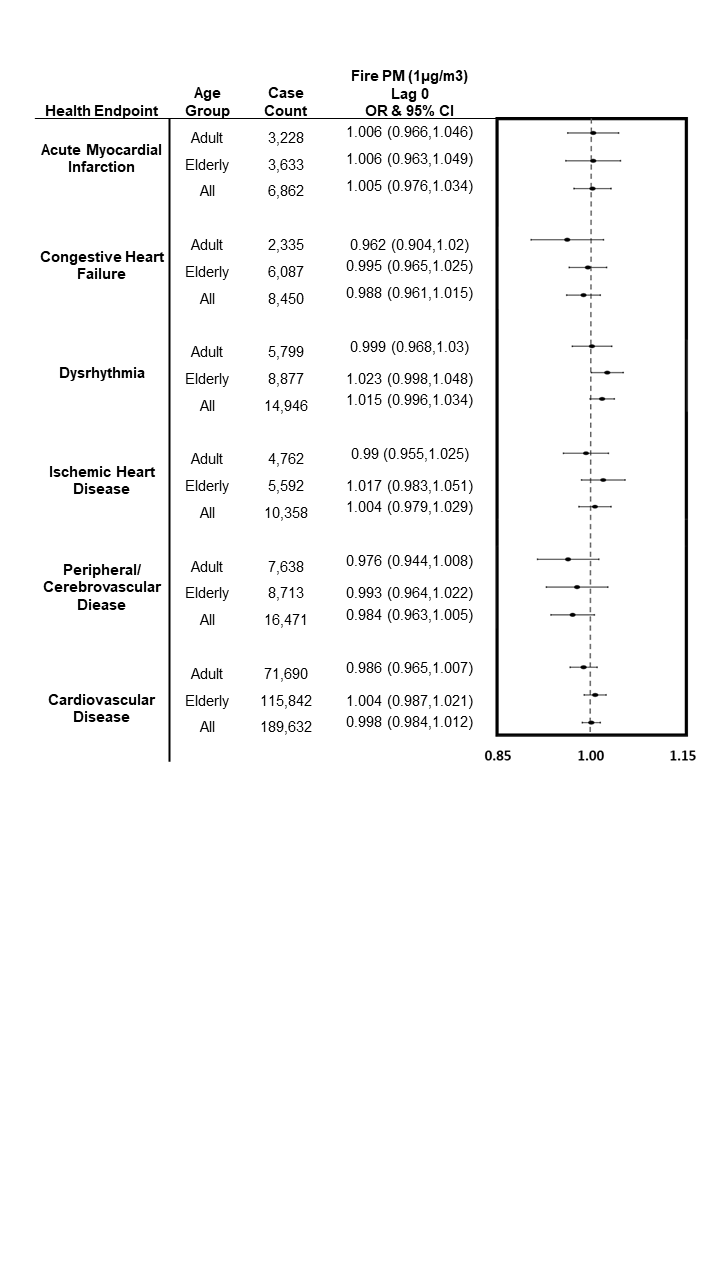


Supplemental Figure 4. OR Cardiovascular Results for Lag 0 Exposures.


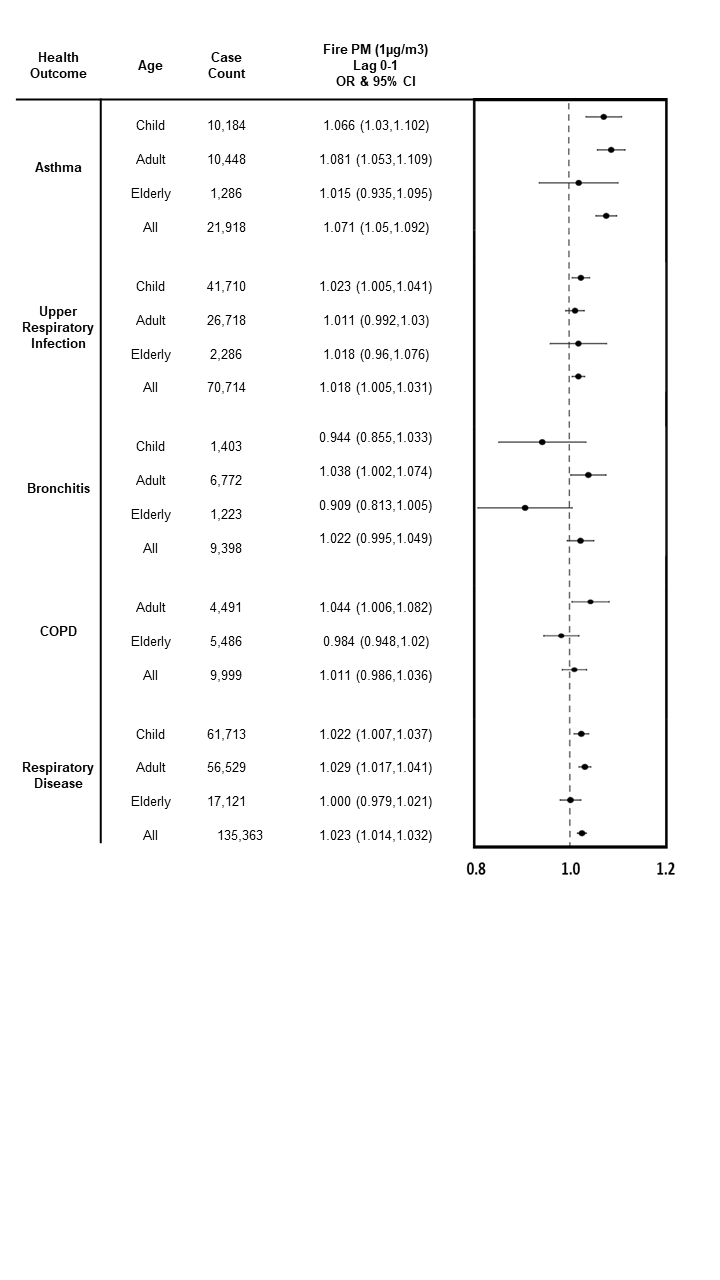
Supplemental Figure 5. OR Respiratory Results for Lag 0-1 Exposures.

| **Supplemental Table 1. Overall Odd Ratios for Respiratory Outcomes due to 3-day average Smoke PM2.5.** | | |
| --- | --- | --- |
| **Health Endpoint** | **Case Count** | **Smoke PM (1µg/m^3^)**  **3-day average**  **OR (95% CI)** |
| **Respiratory** |  |  |
| Asthma | 21,918 | 1.081(1.058, 1.105)* |
|  |  |  |
| Upper respiratory infection | 70,714 | 1.008(0.995, 1.022) |
|  |  |  |
| Bronchitis | 9,398 | 1.018(0.984, 1.052) |
|  |  |  |
| Chronic obstructive pulmonary | 9,999 | 1.020(0.991, 1.049) |
|  |  |  |
| Respiratory disease | 135,363 | 1.021(1.012, 1.031)* |
|  |  |  |

| **Supplemental Table 2. Overall Odd Ratios for Cardiovascular Outcomes due to 2-day average Smoke PM2.5.** | | |
| --- | --- | --- |
| **Health Endpoint** | **Case Count** | **Smoke PM (1µg/m^3^)**  **2-day**  **OR (95% CI)** |
| **Cardiovascular** |  |  |
| Ischemic Heart Disease | 10,358 | 1.011(0.982, 1.041) |
| Acute Myocardial Infarction | 6,862 | 1.006(0.972, 1.042) |
| Dysrhythmia | 14,946 | 1.005(0.982, 1.028) |
| Congestive Heart Failure | 8,450 | 0.986(0.955, 1.018) |
| Peripheral/Cerebrovascular Disease | 16,471 | 0.971(0.946, 0.996) |
| Cardiovascular Disease | 50,225 | 0.998(0.984, 1.011) |

| **Supplemental Table 3. Overall Odd Ratios for Respiratory Outcomes due to 3-day average Total PM2.5.** | | |
| --- | --- | --- |
| **Health Endpoint** | **Case Count** | **Total PM_2.5_ (3µg/m^3^)**  **3-day average**  **OR (95% CI)** |
| **Respiratory** |  |  |
| Asthma | 21,918 | 0.998(0.996, 1.001) |
|  |  |  |
| Upper respiratory infection | 70,714 | 0.998(0.996, 0.999) |
|  |  |  |
| Bronchitis | 9,398 | 1.003(0.998, 1.007) |
|  |  |  |
| Chronic obstructive pulmonary | 9,999 | 0.998(0.994, 1.002) |
|  |  |  |
| Respiratory disease | 135,363 | 0.999(0.998, 1.000) |
|  |  |  |

| **Supplemental Table 4. Overall Odd Ratios for Cardiovascular Outcomes due to 2-day average Total PM2.5.** | | |
| --- | --- | --- |
| **Health Endpoint** | **Case Count** | **Total PM_2.5_ (3µg/m^3^)**  **2-day average**  **OR (95% CI)** |
| **Cardiovascular** |  |  |
| Ischemic Heart Disease | 10,358 | 0.998(0.995, 1.002) |
|  |  |  |
| Acute Myocardial Infarction | 6,862 | 1.002(0.998, 1.007) |
|  |  |  |
| Dysrhythmia | 14,946 | 0.997(0.994, 1.000) |
|  |  |  |
| Congestive Heart Failure | 8,450 | 0.994(0.990, 0.998) |
|  |  |  |
| Peripheral/Cerebrovascular Disease | 16,471 | 0.995(0.992, 0.997) |
|  |  |  |
| Cardiovascular Disease | 189,632 | 0.994(0.993, 0.996) |

| **Supplemental Table 5. Overall Odd Ratios for Respiratory Outcomes due to 7-day average Smoke PM2.5.** | | |
| --- | --- | --- |
| **Health Endpoint** | **Case Count** | **Smoke PM (1µg/m^3^)**  **7-day average**  **OR (95% CI)** |
| **Respiratory** |  |  |
| Asthma | 21,918 | 1.081 (1.051,1.112)* |
|  |  |  |
| Upper respiratory infection | 70,714 | 0.987 (0.970,1.004) |
|  |  |  |
| Bronchitis | 9,398 | 1.029 (0.986,1.074) |
|  |  |  |
| Chronic obstructive pulmonary | 9,999 | 1.022 (0.987,1.059) |
|  |  |  |
| Respiratory disease | 135,363 | 1.007 (0.995,1.019) |
|  |  |  |

| **Supplemental Table 6. Overall Odd Ratios for Respiratory Outcomes due to 7-day average Total PM2.5.** | | |
| --- | --- | --- |
| **Health Endpoint** | **Case Count** | **Total PM_2.5_ (3µg/m^3^)**  **7-day average**  **OR (95% CI)** |
| **Respiratory** |  |  |
| Asthma | 21,918 | 1.001 (0.997,1.004) |
|  |  |  |
| Upper respiratory infection | 70,714 | 0.998 (0.996,1.000) |
|  |  |  |
| Bronchitis | 9,398 | 1.004 (0.998,1.009) |
|  |  |  |
| Chronic obstructive pulmonary | 9,999 | 0.996 (0.991,1.001) |
|  |  |  |
| Respiratory disease | 135,363 | 0.999 (0.998,1.000) |
|  |  |  |

| **Supplemental Table 7. Overall Odd Ratios for Cardiovascular Outcomes due to 3-day average Smoke PM2.5.** | | |
| --- | --- | --- |
| **Health Endpoint** | **Case Count** | **Smoke PM (1µg/m^3^)**  **3-day**  **OR (95% CI)** |
| **Cardiovascular** |  |  |
| Ischemic Heart Disease | 10,358 | 1.011 (0.980,1.043) |
|  |  |  |
| Acute Myocardial Infarction | 6,862 | 1.000 (0.963,1.038) |
|  |  |  |
| Dysrhythmia | 14,946 | 0.995 (0.970,1.021) |
|  |  |  |
| Congestive Heart Failure | 8,450 | 0.979 (0.944,1.015) |
|  |  |  |
| Peripheral/Cerebrovascular Disease | 16,471 | 0.963 (0.937,0.991) |
|  |  |  |
| Cardiovascular Disease | 50,225 | 0.991 (0.976,1.005) |

| **Supplemental Table 8. Overall Odd Ratios for Cardiovascular Outcomes due to 3-day average Total PM2.5.** | | |
| --- | --- | --- |
| **Health Endpoint** | **Case Count** | **Total PM_2.5_ (3µg/m^3^)**  **3-day average**  **OR (95% CI)** |
| **Cardiovascular** |  |  |
| Ischemic Heart Disease | 10,358 | 0.998 (0.994,1.001) |
|  |  |  |
| Acute Myocardial Infarction | 6,862 | 1.001 (0.996,1.006) |
|  |  |  |
| Dysrhythmia | 14,946 | 0.996 (0.993,1.000) |
|  |  |  |
| Congestive Heart Failure | 8,450 | 0.995 (0.990,1.000) |
|  |  |  |
| Peripheral/Cerebrovascular Disease | 16,471 | 0.994 (0.991,0.997) |
|  |  |  |
| Cardiovascular Disease | 189,632 | 0.993 (0.992,0.995) |

| **Supplemental Table 9. Sex Stratified Odd Ratios for Respiratory Outcomes due to 3-day average Total PM2.5.** | | |
| --- | --- | --- |
| **Health Endpoint** | **Case Count** | **Total PM_2.5_ (3µg/m^3^)**  **3-day average**  **OR (95% CI)** |
| **Respiratory** |  |  |
| Asthma |  |  |
| Male | 10,647 | 0.998 (0.994,1.002) |
| Female | 11,271 | 0.999 (0.995, 1.002) |
|  |  |  |
| Upper respiratory infection | 70,714 |  |
| Male | 32,013 | 0.998 (0.995,1.000) |
| Female | 38,701 | 0.998 (0.996,1.000) |
|  |  |  |
| Bronchitis | 9,398 |  |
| Male | 3,675 | 0.997 (0.990,1.005) |
| Female | 5,723 | 1.007 (1.001,1.013)* |
|  |  |  |
| Chronic obstructive pulmonary | 9,999 |  |
| Male | 4,699 | 0.997 (0.991,1.003) |
| Female | 5,300 | 0.999 (0.994,1.005) |
|  |  |  |
| Respiratory disease | 135,363 |  |
| Male | 62,889 | 0.998 (0.997,1.000) |
| Female | 72,474 | 0.999 (0.998,1.001) |
|  |  |  |

| **Supplemental Table 10. Sex Stratified Odd Ratios for Cardiovascular Outcomes due to 2-day average Total PM2.5.** | | |
| --- | --- | --- |
| **Health Endpoint** | **Case Count** | **Total PM (1µg/m^3^)**  **2-day**  **OR (95% CI)** |
| **Cardiovascular** |  |  |
| Ischemic Heart Disease | 10,358 |  |
| Male | 6,893 | 0.998 (0.994,1.003) |
| Female | 3,465 | 0.998 (0.992,1.004) |
|  |  |  |
| Acute Myocardial Infarction | 6,862 |  |
| Male | 4,592 | 1.002 (0.997,1.008) |
| Female | 2,270 | 1.002 (0.995,1.010) |
|  |  |  |
| Dysrhythmia | 14,946 |  |
| Male | 7,615 | 0.997 (0.993,1.001) |
| Female | 7,331 | 0.997 (0.992,1.001) |
|  |  |  |
| Congestive Heart Failure | 8,450 |  |
| Male | 4,262 | 0.996 (0.991,1.002) |
| Female | 4,188 | 0.992 (0.986,0.998) |
|  |  |  |
| Peripheral/Cerebrovascular Disease | 16,471 |  |
| Male | 7,816 | 0.994 (0.990,0.998) |
| Female | 8,655 | 0.995 (0.991,0.999) |
|  |  |  |
| Cardiovascular Disease | 50,225 |  |
| Male | 26,586 | 0.995 (0.993,0.997) |
| Female | 23,639 | 0.994 (0.991,0.996) |

| **Supplemental Table 11. Sex Stratified Odd Ratios for Cardiovascular Outcomes due to 2-day Smoke PM2.5.** | | |
| --- | --- | --- |
| **Health Endpoint** | **Case Count** | **Smoke PM (1µg/m^3^)**  **2-day**  **OR (95% CI)** |
| **Cardiovascular** |  |  |
| Ischemic Heart Disease | 10,358 |  |
| Male | 6,893 | 0.997 (0.961,1.033) |
| Female | 3,465 | 1.038 (0.989,1.090) |
|  |  |  |
| Acute Myocardial Infarction | 6,862 |  |
| Male | 4,592 | 1.004 (0.963,1.048) |
| Female | 2,270 | 1.010 (0.952,1.072) |
|  |  |  |
| Dysrhythmia | 14,946 |  |
| Male | 7,615 | 1.011 (0.981,1.043) |
| Female | 7,331 | 0.997 (0.964,1.032) |
|  |  |  |
| Congestive Heart Failure | 8,450 |  |
| Male | 4,262 | 0.991 (0.946,1.039) |
| Female | 4,188 | 0.982 (0.939,1.026) |
|  |  |  |
| Peripheral/Cerebrovascular Disease | 16,471 |  |
| Male | 7,816 | 0.958 (0.922,0.996) |
| Female | 8,655 | 0.981 (0.948,1.016) |
|  |  |  |
| Cardiovascular Disease | 50,225 |  |
| Male | 26,586 | 0.997 (0.979,1.016) |
| Female | 23,639 | 0.997 (0.978,1.017) |

| **Supplemental Table 12. Sex Stratified Odd Ratios for Respiratory Outcomes due to 3-day average Smoke PM2.5.** | | |
| --- | --- | --- |
| **Health Endpoint** | **Case Count** | **Smoke PM_2.5_ (3µg/m^3^)**  **3-day average**  **OR (95% CI)** |
| **Respiratory** |  |  |
| Asthma |  |  |
| Male | 10,647 | 1.063 (1.029,1.098)* |
| Female | 11,271 | 1.096 (1.064,1.128)* |
|  |  |  |
| Upper respiratory infection |  |  |
| Male | 32,013 | 1.000 (0.980,1.021) |
| Female | 38,701 | 1.015 (0.997,1.034) |
|  |  |  |
| Bronchitis |  |  |
| Male | 3,675 | 0.970 (0.920,1.023) |
| Female | 5,723 | 1.054 (1.010,1.101)* |
|  |  |  |
| Chronic obstructive pulmonary |  |  |
| Male | 4,699 | 1.019 (0.978,1.061) |
| Female | 5,300 | 1.020 (0.981,1.061) |
|  |  |  |
| Respiratory disease |  |  |
| Male | 62,889 | 1.014 (1.000,1.028) |
| Female | 72,474 | 1.027 (1.015,1.040)* |
|  |  |  |
|  |  |  |

| **Supplemental Table 13. Age Stratified Odd Ratios for Respiratory Outcomes due to 3-day average Total PM2.5.** | | |
| --- | --- | --- |
| **Health Endpoint** | **Case Count** | **Total PM_2.5_ (3µg/m^3^)**  **3-day average**  **OR (95% CI)** |
| **Respiratory** |  |  |
| Asthma |  |  |
| Children (0-18) | 10,184 | 0.998 (0.994,1.002) |
| Adults (19-64) | 10,448 | 0.999 (0.995, 1.003) |
| Older Adult (65+) | 1,286 | 1.000 (0.988, 1.012) |
|  |  |  |
| Upper respiratory infection |  |  |
| Children (0-18) | 41,710 | 0.999 (0.997,1.001) |
| Adults (19-64) | 26,718 | 0.995 (0.993,0.999) |
| Older Adult (65+) | 2,286 | 0.998 (0.990,1.007) |
|  |  |  |
| Bronchitis |  |  |
| Children (0-18) | 1,403 | 1.008 (0.996,1.020) |
| Adults (19-64) | 6,772 | 1.003 (0.997,1.008) |
| Older Adult (65+) | 1,223 | 0.999 (0.986,1.011) |
|  |  |  |
| Chronic obstructive pulmonary |  |  |
| Children (0-18) | 22 | -- |
| Adults (19-64) | 4,491 | 1.000 (0.994,1.006) |
| Older Adult (65+) | 5,486 | 0.997 (0.991,1.002) |
|  |  |  |
| Respiratory disease |  |  |
| Children (0-18) | 61,713 | 1.001 (0.999,1.002) |
| Adults (19-64) | 56,529 | 0.998 (0.996,1.000) |
| Older Adult (65+) | 17,121 | 0.996 (0.993,0.999) |
|  |  |  |

| **Supplemental Table 14. Age Stratified Odd Ratios for Respiratory Outcomes due to 3-day average Smoke PM2.5.** | | |
| --- | --- | --- |
| **Health Endpoint** | **Case Count** | **Smoke PM (1µg/m^3^)**  **3-day average**  **OR (95% CI)** |
| **Respiratory** |  |  |
| Asthma |  |  |
| Children (0-18) | 10,184 | 1.075 (1.035,1.116)* |
| Adults (19-64) | 10,448 | 1.091 (1.060, 1.122)* |
| Older Adult (65+) | 1,286 | 1.009 (0.920, 1.106) |
|  |  |  |
| Upper respiratory infection |  |  |
| Children (0-18) | 41,710 | 1.010 (0.991,1.029) |
| Adults (19-64) | 26,718 | 1.005 (0.984,1.026) |
| Older Adult (65+) | 2,286 | 1.004 (0.940,1.072) |
|  |  |  |
| Bronchitis |  |  |
| Children (0-18) | 1,403 | 0.971 (0.890,1.060) |
| Adults (19-64) | 6,772 | 1.044 (1.005,1.085)* |
| Older Adult (65+) | 1,223 | 0.908 (0.805,1.024) |
|  |  |  |
| Chronic obstructive pulmonary |  |  |
| Children (0-18) | 22 | -- |
| Adults (19-64) | 4,491 | 1.056 (1.015,1.100)* |
| Older Adult (65+) | 5,486 | 0.989 (0.951,1.030) |
|  |  |  |
| Respiratory disease |  |  |
| Children (0-18) | 61,713 | 1.016 (1.000,1.032) |
| Adults (19-64) | 56,529 | 1.030 (1.017,1.044)* |
| Older Adult (65+) | 17,121 | 1.000 (0.976,1.024) |
|  |  |  |

| **Supplemental Table 15. Age Stratified Odd Ratios for Cardiovascular Outcomes due to 2-day average Total PM2.5.** | | |
| --- | --- | --- |
| **Health Endpoint** | **Case Count** | **Total PM_2.5_ (3µg/m^3^)**  **2-day average**  **OR (95% CI)** |
| **Cardiovascular** |  |  |
| Ischemic Heart Disease |  |  |
| Adults (19-64) | 4,762 | 1.000 (0.995,1.005) |
| Elderly (65+) | 5,592 | 0.997 (0.992,1.002) |
|  |  |  |
| Acute Myocardial Infarction |  |  |
| Adults (19-64) | 3,228 | 1.005 (0.999,1.011) |
| Elderly (65+) | 3,633 | 1.000 (0.994,1.006) |
|  |  |  |
| Dysrhythmia |  |  |
| Adults (19-64) | 5,799 | 0.999 (0.994,1.004) |
| Elderly (65+) | 8,877 | 0.996 (0.992,1.000) |
|  |  |  |
| Congestive Heart Failure |  |  |
| Adults (19-64) | 2,335 | 0.991 (0.983,0.999) |
| Elderly (65+) | 6,087 | 0.996 (0.991,1.000) |
|  |  |  |
| Peripheral/Cerebrovascular Disease |  |  |
| Adults (19-64) | 7,638 | 0.994 (0.990,0.998) |
| Elderly (65+) | 8,713 | 0.995 (0.991,0.999) |
|  |  |  |
| Cardiovascular Disease |  |  |
| Adults (19-64) | 71,690 | 0.995 (0.992,0.998) |
| Elderly (65+) | 115,842 | 0.994 (0.992,0.996) |

| **Supplemental Table 16. Age Stratified Odd Ratios for Cardiovascular Outcomes due to 2-day average Smoke PM2.5.** | | |
| --- | --- | --- |
| **Health Endpoint** | **Case Count** | **Smoke PM (1µg/m^3^)**  **2-day**  **OR (95% CI)** |
| **Cardiovascular** |  |  |
| Ischemic Heart Disease |  |  |
| Adults (19-64) | 4,762 | 0.999 (0.957, 1.043) |
| Elderly (65+) | 5,592 | 1.022 (0.982, 1.063) |
|  |  |  |
| Acute Myocardial Infarction |  |  |
| Adults (19-64) | 3,228 | 1.011 (0.963,1.062) |
| Elderly (65+) | 3,633 | 1.002 (0.954,1.052) |
|  |  |  |
| Dysrhythmia |  |  |
| Adults (19-64) | 5,799 | 0.993(0.956,1.030) |
| Elderly (65+) | 8,877 | 1.010 (0.980,1.040) |
|  |  |  |
| Congestive Heart Failure |  |  |
| Adults (19-64) | 2,335 | 0.962 (0.896,1.032) |
| Elderly (65+) | 6,087 | 0.993 (0.957,1.029) |
|  |  |  |
| Peripheral/Cerebrovascular Disease |  |  |
| Adults (19-64) | 7,638 | 0.964 (0.928,1.002) |
| Elderly (65+) | 8,713 | 0.978 (0.945,1.013) |
|  |  |  |
| Cardiovascular Disease |  |  |
| Adults (19-64) | 20,534 | 0.986 (0.965,1.008) |
| Elderly (65+) | 29,269 | 1.004 (0.987,1.021) |

| **Supplemental Table 17. Age Stratified Odd Ratios for Respiratory Outcomes due to 7-day average Smoke PM2.5.** | | |
| --- | --- | --- |
| **Health Endpoint** | **Case Count** | **Smoke PM (1µg/m^3^)**  **7-day average**  **OR (95% CI)** |
| **Respiratory** |  |  |
| Asthma |  |  |
| Children (0-18) | 10,184 | 1.053 (1.001,1.108)* |
| Adults (19-64) | 10,448 | 1.097 (1.059, 1.138)* |
| Older Adult (65+) | 1,286 | 1.023 (0.912, 1.148) |
|  |  |  |
| Upper respiratory infection |  |  |
| Children (0-18) | 41,710 | 0.980 (0.957,1.005) |
| Adults (19-64) | 26,718 | 0.995 (0.970,1.021) |
| Older Adult (65+) | 2,286 | 0.961 (0.879,1.049) |
|  |  |  |
| Bronchitis |  |  |
| Children (0-18) | 1,403 | 1.017 (0.922,1.122) |
| Adults (19-64) | 6,772 | 1.046 (0.994,1.101) |
| Older Adult (65+) | 1,223 | 0.935 (0.809,1.080) |
|  |  |  |
| Chronic obstructive pulmonary |  |  |
| Children (0-18) | 22 | -- |
| Adults (19-64) | 4,491 | 1.059 (1.005,1.115)* |
| Older Adult (65+) | 5,486 | 0.994 (0.946,1.045) |
|  |  |  |
| Respiratory disease |  |  |
| Children (0-18) | 61,713 | 0.990 (0.970,1.010) |
| Adults (19-64) | 56,529 | 1.019 (1.002,1.036)* |
| Older Adult (65+) | 17,121 | 0.999 (0.970,1.029) |
|  |  |  |

| **Supplemental Table 18. Age Stratified Odd Ratios for Respiratory Outcomes due to 7-day average Total PM2.5.** | | |
| --- | --- | --- |
| **Health Endpoint** | **Case Count** | **Total PM_2.5_ (3µg/m^3^)**  **7-day average**  **OR (95% CI)** |
| **Respiratory** |  |  |
| Asthma |  |  |
| Children (0-18) | 10,184 | 1.000 (0.994,1.005) |
| Adults (19-64) | 10,448 | 1.002 (0.997, 1.007) |
| Older Adult (65+) | 1,286 | 0.997 (0.983, 1.012) |
|  |  |  |
| Upper respiratory infection | 70,714 |  |
| Children (0-18) | 41,710 | 0.999 (0.996,1.001) |
| Adults (19-64) | 26,718 | 0.997 (0.994,1.000) |
| Older Adult (65+) | 2,286 | 1.003 (0.993,1.014) |
|  |  |  |
| Bronchitis | 9,398 |  |
| Children (0-18) | 1,403 | 1.006 (0.991,1.022) |
| Adults (19-64) | 6,772 | 1.003 (0.996,1.010) |
| Older Adult (65+) | 1,223 | 1.004 (0.989,1.020) |
|  |  |  |
| Chronic obstructive pulmonary | 9,999 |  |
| Children (0-18) | 22 | -- |
| Adults (19-64) | 4,491 | 0.998 (0.991,1.006) |
| Older Adult (65+) | 5,486 | 0.994 (0.988,1.001) |
|  |  |  |
| Respiratory disease | 135,363 |  |
| Children (0-18) | 61,713 | 1.000 (0.998,1.002) |
| Adults (19-64) | 56,529 | 0.999 (0.997,1.001) |
| Older Adult (65+) | 17,121 | 0.995 (0.991,1.000) |
|  |  |  |

| **Supplemental Table 19. Sex Stratified Odd Ratios for Respiratory Outcomes due to 7-day average Smoke PM2.5.** | | |
| --- | --- | --- |
| **Health Endpoint** | **Case Count** | **Smoke PM_2.5_ (3µg/m^3^)**  **7-day average**  **OR (95% CI)** |
| **Respiratory** |  |  |
| Asthma |  |  |
| Male | 10,647 | 1.071 (1.027,1.117)* |
| Female | 11,271 | 1.089 (1.048,1.131)* |
|  |  |  |
| Upper respiratory infection | 70,714 |  |
| Male | 32,013 | 0.973 (0.948,0.999) |
| Female | 38,701 | 0.998 (0.975,1.021) |
|  |  |  |
| Bronchitis | 9,398 |  |
| Male | 3,675 | 0.994 (0.930,1.063) |
| Female | 5,723 | 1.055 (0.998,1.116) |
|  |  |  |
| Chronic obstructive pulmonary | 9,999 |  |
| Male | 4,699 | 1.018 (0.967,1.071) |
| Female | 5,300 | 1.026 (0.977,1.078) |
|  |  |  |
| Respiratory disease | 135,363 |  |
| Male | 62,889 | 1.004 (0.987,1.022) |
| Female | 72,474 | 1.010 (0.994,1.026) |
|  |  |  |
|  |  |  |

| **Supplemental Table 20. Sex Stratified Odd Ratios for Respiratory Outcomes due to 7-day average Total PM2.5.** | | |
| --- | --- | --- |
| **Health Endpoint** | **Case Count** | **Total PM_2.5_ (3µg/m^3^)**  **7-day average**  **OR (95% CI)** |
| **Respiratory** |  |  |
| Asthma |  |  |
| Male | 10,647 | 1.000 (0.995,1.005) |
| Female | 11,271 | 1.001 (0.997,1.006) |
|  |  |  |
| Upper respiratory infection |  |  |
| Male | 32,013 | 0.999 (0.996,1.001) |
| Female | 38,701 | 0.998 (0.995,1.000) |
|  |  |  |
| Bronchitis |  |  |
| Male | 3,675 | 1.000 (0.991,1.009) |
| Female | 5,723 | 1.006 (0.999,1.014) |
|  |  |  |
| Chronic obstructive pulmonary |  |  |
| Male | 4,699 | 0.994 (0.987,1.001) |
| Female | 5,300 | 0.998 (0.991,1.004) |
|  |  |  |
| Respiratory disease |  |  |
| Male | 62,889 | 0.999 (0.997,1.001) |
| Female | 72,474 | 0.999 (0.997,1.001) |
|  |  |  |

| **Supplemental Table 21. Age Stratified Odd Ratios for Cardiovascular Outcomes due to 3-day average Smoke PM2.5.** | | |
| --- | --- | --- |
| **Health Endpoint** | **Case Count** | **Smoke PM (1µg/m^3^)**  **3-day**  **OR (95% CI)** |
| **Cardiovascular** |  |  |
| Ischemic Heart Disease | 10,358 |  |
| Adults (19-64) | 4,762 | 1.011 (0.966, 1.059) |
| Elderly (65+) | 5,592 | 1.010 (0.968, 1.055) |
|  |  |  |
| Acute Myocardial Infarction | 6,862 |  |
| Adults (19-64) | 3,228 | 1.016 (0.964,1.071) |
| Elderly (65+) | 3,633 | 0.985 (0.935,1.037) |
|  |  |  |
| Dysrhythmia | 14,946 |  |
| Adults (19-64) | 5,799 | 0.986(0.946,1.027) |
| Elderly (65+) | 8,877 | 0.997 (0.965,1.031) |
|  |  |  |
| Congestive Heart Failure | 8,450 |  |
| Adults (19-64) | 2,335 | 0.974 (0.906,1.048) |
| Elderly (65+) | 6,087 | 0.980 (0.940,1.022) |
|  |  |  |
| Peripheral/Cerebrovascular Disease | 16,471 |  |
| Adults (19-64) | 7,638 | 0.952 (0.912,0.993) |
| Elderly (65+) | 8,713 | 0.973 (0.937,1.011) |
|  |  |  |
| Cardiovascular Disease | 50,225 |  |
| Adults (19-64) | 20,534 | 0.984 (0.961,1.007) |
| Elderly (65+) | 29,269 | 0.994 (0.975,1.013) |

| **Supplemental Table 22. Age Stratified Odd Ratios for Cardiovascular Outcomes due to 3-day average Total PM2.5.** | | |
| --- | --- | --- |
| **Health Endpoint** | **Case Count** | **Total PM_2.5_ (3µg/m^3^)**  **3-day average**  **OR (95% CI)** |
| **Cardiovascular** |  |  |
| Ischemic Heart Disease | 10,358 |  |
| Adults (19-64) | 4,762 | 1.000 (0.995, 1.006) |
| Elderly (65+) | 5,592 | 0.995 (0.990, 1.000) |
|  |  |  |
| Acute Myocardial Infarction | 6,862 |  |
| Adults (19-64) | 3,228 | 1.005 (0.998,1.012) |
| Elderly (65+) | 3,633 | 0.998 (0.991,1.005) |
|  |  |  |
| Dysrhythmia | 14,946 |  |
| Adults (19-64) | 5,799 | 0.997 (0.992,1.003) |
| Elderly (65+) | 8,877 | 0.995 (0.991,0.999) |
|  |  |  |
| Congestive Heart Failure | 8,450 |  |
| Adults (19-64) | 2,335 | 0.993 (0.985,1.002) |
| Elderly (65+) | 6,087 | 0.995 (0.990,1.001) |
|  |  |  |
| Peripheral/Cerebrovascular Disease | 16,471 |  |
| Adults (19-64) | 7,638 | 0.992 (0.988,0.997) |
| Elderly (65+) | 8,713 | 0.995 (0.991,0.999) |
|  |  |  |
| Cardiovascular Disease | 189,632 |  |
| Adults (19-64) | 71,690 | 0.994 (0.991,0.997) |
| Elderly (65+) | 115,842 | 0.993 (0.991,0.995) |

| **Supplemental Table 23. Sex Stratified Odd Ratios for Cardiovascular Outcomes due to 3-day average Smoke PM2.5.** | | |
| --- | --- | --- |
| **Health Endpoint** | **Case Count** | **Smoke PM (1µg/m^3^)**  **3-day**  **OR (95% CI)** |
| **Cardiovascular** |  |  |
| Ischemic Heart Disease | 10,358 |  |
| Male | 6,893 | 0.999 (0.960,1.038) |
| Female | 3,465 | 1.034 (0.981,1.090) |
|  |  |  |
| Acute Myocardial Infarction | 6,862 |  |
| Male | 4,592 | 0.998 (0.953,1.044) |
| Female | 2,270 | 1.003 (0.941,1.069) |
|  |  |  |
| Dysrhythmia | 14,946 |  |
| Male | 7,615 | 1.002 (0.968,1.038) |
| Female | 7,331 | 0.987 (0.951,1.025) |
|  |  |  |
| Congestive Heart Failure | 8,450 |  |
| Male | 4,262 | 1.000 (0.950,1.054) |
| Female | 4,188 | 0.961 (0.913,1.011) |
|  |  |  |
| Peripheral/Cerebrovascular Disease | 16,471 |  |
| Male | 7,816 | 0.956 (0.917,0.966) |
| Female | 8,655 | 0.970 (0.933,1.008) |
|  |  |  |
| Cardiovascular Disease | 50,225 |  |
| Male | 26,586 | 0.995 (0.975,1.015) |
| Female | 23,639 | 0.986 (0.965,1.007) |

| **Supplemental Table 24. Sex Stratified Odd Ratios for Cardiovascular Outcomes due to 3-day average Total PM2.5.** | | |
| --- | --- | --- |
| **Health Endpoint** | **Case Count** | **Total PM (1µg/m^3^)**  **3-day**  **OR (95% CI)** |
| **Cardiovascular** |  |  |
| Ischemic Heart Disease | 10,358 |  |
| Male | 6,893 | 0.998 (0.994,1.003) |
| Female | 3,465 | 0.996 (0.989,1.003) |
|  |  |  |
| Acute Myocardial Infarction | 6,862 |  |
| Male | 4,592 | 1.002 (0.996,1.008) |
| Female | 2,270 | 1.000 (0.991,1.008) |
|  |  |  |
| Dysrhythmia | 14,946 |  |
| Male | 7,615 | 0.996 (0.992,1.001) |
| Female | 7,331 | 0.995 (0.991,1.000) |
|  |  |  |
| Congestive Heart Failure | 8,450 |  |
| Male | 4,262 | 0.996 (0.990,1.002) |
| Female | 4,188 | 0.994 (0.987,1.000) |
|  |  |  |
| Peripheral/Cerebrovascular Disease | 16,471 |  |
| Male | 7,816 | 0.993 (0.989,0.998) |
| Female | 8,655 | 0.994 (0.990,0.999) |
|  |  |  |
| Cardiovascular Disease | 50,225 |  |
| Male | 26,586 | 0.994 (0.992,0.996) |
| Female | 23,639 | 0.993 (0.990,0.995) |
